# Accelerated clinical response achieved by combining short-term tumor-directed photodynamic therapy with immunotherapy-based systemic therapies in synchronous colorectal cancer with MSI-H and POLE mutation: a case report

**DOI:** 10.3389/fimmu.2024.1402334

**Published:** 2024-06-28

**Authors:** Yuhan Wang, Lei Gao, Bin Ma, Jianming Shi, Zhenyu Yin, Weidong Zhu, Hao Chen

**Affiliations:** ^1^ Lanzhou University Second Hospital, Lanzhou, China; ^2^ Department of General Surgery, Lintao County People’s Hospital in Gansu Province, Lintao, China; ^3^ Department of Surgical Oncology, Gansu Provincial Key Laboratory Of Environmental Oncology, Lanzhou University Second Hospital, Lanzhou, China

**Keywords:** colorectal neoplasms, immunotherapy, photodynamic therapy, microsatellite instability, POLE protein, human

## Abstract

Genetic sequencing has revolutionized immunotherapy in colorectal cancer (CRC). Recent clinical trials have revealed a positive response to immunotherapy-based systemic therapies in CRC patient subgroups with microsatellite instability (MSI)-High or DNA polymerase epsilon (POLE) mutation. However, the unsatisfactory response rates was the major limitation in real-world practice of the precision immunotherapy in CRC. Adding photodynamic therapy (PDT) to systemic immunotherapy has showed synergetic anti-tumor effect by modulating tumor microenvironment, while the eligible patient’s subgroups which would benefit from this combination remained equivocal. Here we reported a synchronous colorectal cancer patient with MSI-High and POLE mutation who had accelerated response in less than 2 cycles (42 days) of immunotherapy-based systemic therapies after tumor-directed PDT and has remained progression-free by far. This case enlightened the synergetic effect of PDT in immunotherapy-treated CRC patients, with the MSI and POLE-mutation status as predictors of survival benefits.

## Introduction

Colorectal cancer (CRC) has emerged as a major threat to global health. Global epidemic statistics have reported that the incidence of colorectal cancer worldwide has increased through the last decade and ranked 3^rd^ in all cancer types (1). In China, both the incidence and the mortality rate of colorectal cancer kept increasing (2). Contemporary treatment modalities in localized CRC were surgery-centered, while other local treatment modalities remained only complementary or palliative. However, the enormous surgery-related complications and the drastic destructions on life quality caused by surgery were unacceptable for quite a lot of CRC patients (3). For patients who refused to receive surgery, systemic therapy was widely regarded as palliative. The immunotherapy-based systemic approach has been recommended in colorectal cancer management, especially for patients with unique gene mutation features like MSI-H or POLE (4). Recently Chen et al. (5) reported an encouraging result from a clinical trial where 9 of 17 locally-advanced rectal cancer patients got a complete response after 4 cycles of neoadjuvant PD-1 inhibitor sintilimab monotherapy, which indicated the possibility of achieving complete clinical response in MSI-H CRC patients without undertaken surgical interventions. To optimize the therapeutic effect and minimize the time to clinical response, the incorporation of local therapies like photodynamic therapy (PDT) with systemic therapy was thought to improve the local tumor control in CRC patients who refused to undergo surgical procedures (4). However, the combinations of PDT with immunotherapy remained only exploratory. This report presented a case of locally-advanced synchronous colorectal cancer patient with MSI-H and POLE mutation and high tumor burden who got accelerated clinical response after combining short-term photodynamic therapy and immunotherapy-based systemic therapies and has remained progression-free by far.

## Case description

**Figure 1:**
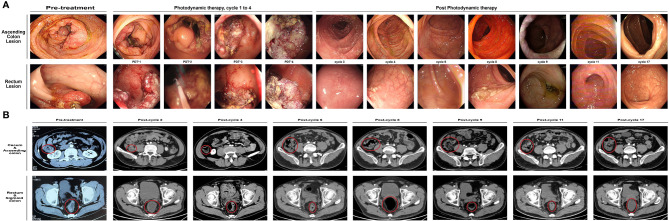
Visualizations of treatment efficacy in this patient. **(A)** Tumor lesions in the ascending colon and the rectum under coloscopy during the treatment. All lesions showed significant necrotic changes with photodynamic therapy. After the local photodynamic therapy, no tumor residual and local recurrence were observed under coloscopy through these cycles. **(B)** The patient’s abdominal CT scan images in cecum/ascending colon and rectum (indicated by red circles) pre-treatment, post cycle-2, post-cycle-4, post cycle-6, post cycle-8, post cycle-9, post cycle-11 and post cycle-17, respectively.

**Figure 2:**
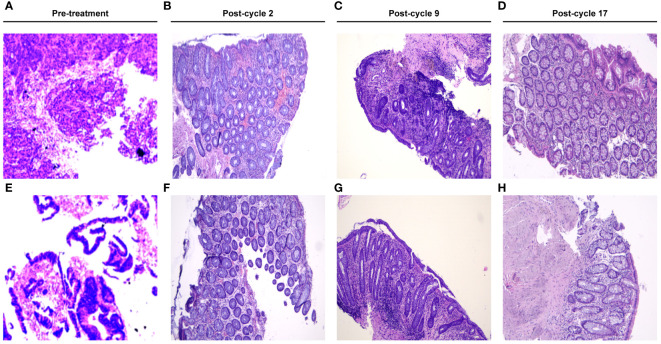
Microscopic view of the hematoxylin-eosin (HE)-stained biopsy in this patient. **(A, E)** showed the microscopic view of the HE-stained tumor biopsy at the 1st admission. **(B–D, F–H)** revealed the chronic inflammation change without tumor residual post cycle-2, post cycle-9 and post cycle-17, respectively.

A 40-year-old Asian male patient presented with abdominal pain and melena for 10 days was admitted to our hospital on July 4^th^, 2022. Physical examinations at admission were negative except for a mass located around 5 cm away from the anus. After admission, the patient was ordered enhanced contrast abdominal CT scan as well as the coloscopy. The thickening of ascending colon and upper medium rectum, the lumen stricture of the affected colon, with the invasion of serosa membrane and the peri-intestinal adipose tissues were observed from the abdominal CT (**Figure 1B**). The coloscopy revealed that two protuberant ulcerated masses located 6 cm and 15cm away from the anus, an ulcerated mass in the ileocecal valve and a huge polypoid fungating nodular mass that almost occulted the lumen of ascending colon (**Figure 1A**). Multiple biopsies were obtained from the abovementioned masses and were sent for the pathological examinations, which revealed that the lesions in the ileocecal valve and ascending colon were middle-differentiated adenocarcinoma while the lesions in the rectum were middle-to-high-differentiated adenocarcinoma (**Figure 2**). The Next-Generation Sequencing (NGS) results and immunohistochemistry analysis were illustrated in **Supplementary Material**, which demonstrated microsatellite instability (MSI)-High (H) and Tumor mutation burden (TMB)-H in this patient. Mutations in immunotherapy-related gene panels like POLE and POLD1 both tested positive, while the expressions of PD-L1 in tumor lesions were negative. To differentiate from hereditary lynch syndrome, mismatch repair (MMR) and BRAF mutations were tested and no mutations were detected, which ensured the diagnosis of sporadic MSI-H CRC. Moreover, the expressions of HER-2 in all biopsies were not amplified. Based on these results, the patient was diagnosed with synchronous colon cancer (sCRC) (for lesions in ileocecal valve and rectum, cT_3_N_1_Mx stage III, for lesion in ascending colon, cT_2_N_1_M_0_ stage III, respectively). Neoadjuvant systemic therapy followed by pan-colectomy were scheduled for this patient, however the patient refused to receive surgical procedure due to concerns of surgery-related complications. After careful discussions with our multi-disciplinary team and the patient’s family, the patient was enrolled in a clinical study with informed consent. As requested by the protocol of the study (ChiCTR2200064280), this patient would receive PDT together with immunotherapy-based systemic therapy as palliative treatment. In the first treatment cycle, this patient received 4 times PDT under coloscopy followed by systemic therapy from July 16^th^, 2022 to July 18^th^, 2022. The systemic therapy regimen was set to be a combination of CAPOX (capecitabine 1000mg/m^2^ + oxaliplatin 130mg/m^2^), VEGF inhibitor bevacizumab 7.5mg/kg, and PD-1 inhibitor sintilimab 200mg. Every treatment cycle was set to be 21 days. After the first treatment cycle, this patient was discharged from the hospital with no significant treatment-related adverse events. This patient continued to receive the following 2^nd^ to 8^th^ cycle immunotherapy-based systemic therapy with the same regimen. After the 8^th^ treatment cycle, the patient has refused chemotherapy. Therefore, all chemotherapy drugs have been excluded from the treatment plan in the following 9^th^ to 17^th^ cycle. The current palliative regimen for this patient was merely immunotherapy and targeted therapy.

**Figure 3:**
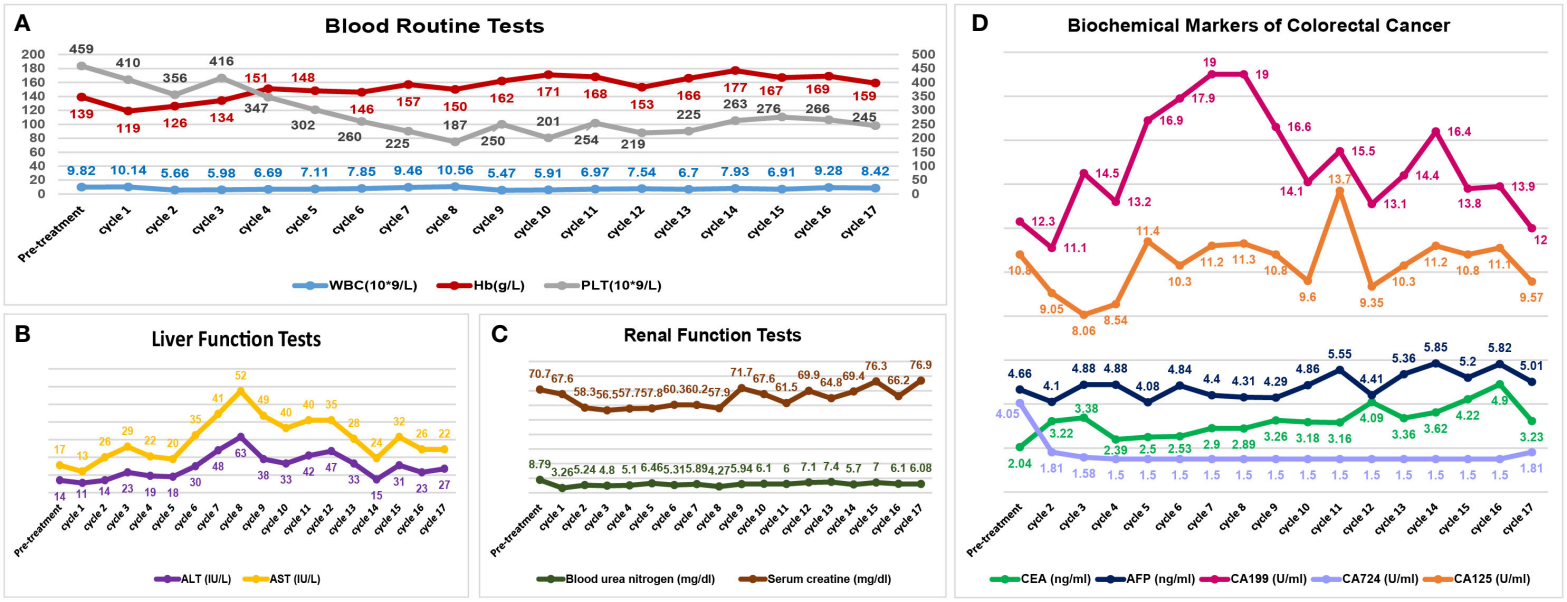
Visualizations of safety profiles in this patient. The blood tests **(A)**, liver function tests **(B)** and renal function tests **(C)** have remained stable through these treatment cycles. The biochemical markers of colorectal cancer **(D)** have also been illustrated. (WBC, white blood cell count; Hb, hemoglobin; PLT, blood platelet count; ALT, alanine aminotransferase; AST, aspartate aminotransferase; CEA, carcinoembryonic antigen; AFP, α-fetoprotein).

Efficacy was evaluated every two cycles. After 2 treatment cycles, both the coloscopy and CT scan of the abdomen demonstrated that the masses in the rectal and colon were completely remitted, with only fibrosis left (**Figure 2**). No sign of local recurrence or distant metastasis was detected in the following treatment cycles. The biopsies of the lesions in previous endoscopic tumor regions proved to be fibrosis and mild inflammatory cell infiltrations (**Figure 2**). After 17 treatment cycles, this patient was assessed as progression-free and in good general condition, with no severe adverse events observed. The results of blood routine tests, liver function tests, renal function tests and the CRC biomarkers through these 17 treatment cycles were illustrated in **Figure 3**.

After the treatment cycle 8, this patient hasn’t undergone the follow-ups regularly due to economic concerns. The progression-free survival in this patient was 21 months up to March 2024.

## Discussion

**Figure 4:**
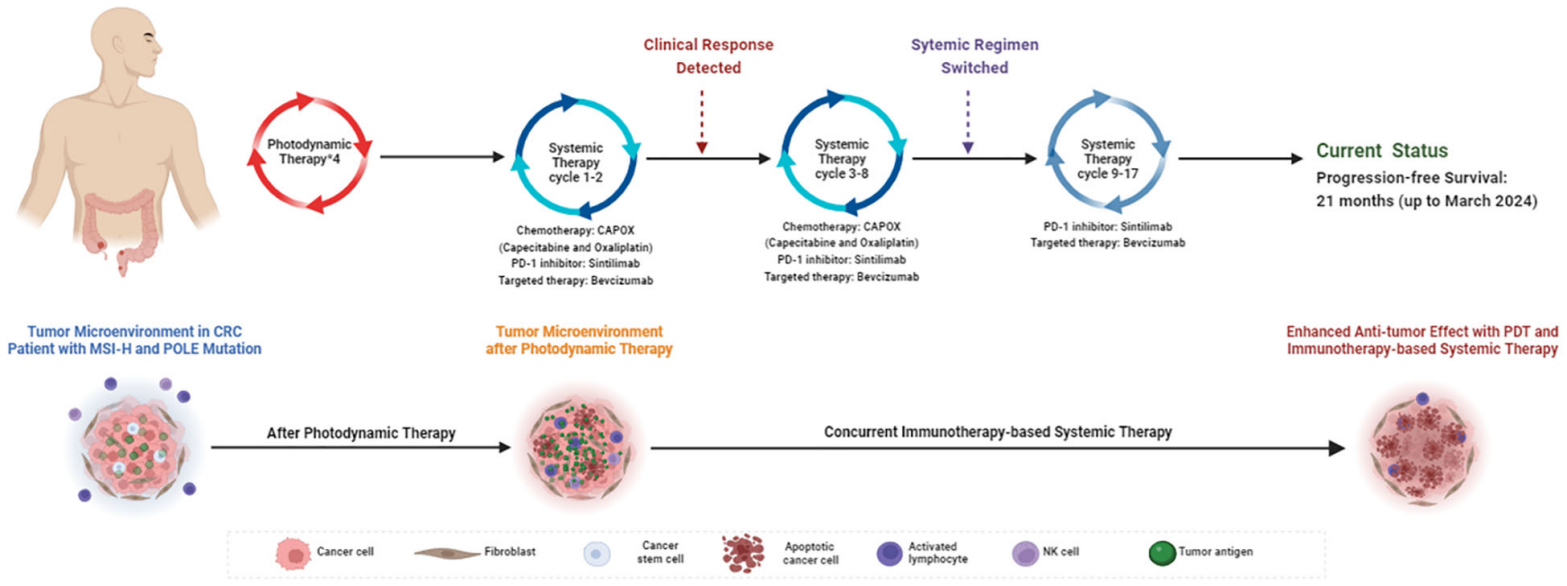
Schematic view of treatment timeline and the potential mechanism of the enhanced anti-tumor effect induced by combining PDT and immunotherapy-based systemic therapy in this case. Created with BioRender.com. (CRC, colorectal cancer; MSI-H, Microsatellite Instability-High; PDT, photodynamic therapy).

PDT was a non-surgical intervention that has been applied in various malignancies (6). Besides generating cytotoxic reactive oxygen species (7), PDT would promote the release of tumor antigens to potentiate the anti-tumor effect of immunotherapy (8). Retrospective studies have revealed that PDT was safe and efficient in stage III/IV CRC patients (9), however its use in real-world clinical practice remained exploratory. In this case, we found that PDT was well-tolerated and has exhibited great efficacy in synchronous CRC, especially in patients with MSI-H and POLE mutation, which would hypothetically synergize the immunotherapy-based systemic therapy (**Figure 4**).

Genomic sequencing has reshaped the landscape of CRC treatments towards individualized management. The immunotherapy-based systemic approach has been recommended in the management of MSI-H-related malignancies by NCCN guidelines (4). In this patient, the existence of MSI-H has been confirmed by the NGS of tumor biopsies. Previous retrospective studies have also revealed that sCRC was featured with a high prevalence of MSI-H status and a high tumor mutation burden (10, 11). The unresponsive rate of immunotherapy in real-world studies was roughly 45% to 60% in CRC patients with MSI-H (12, 13). The resistance mechanism was thought to be related to the absence of tumor antigens in the tumor microenvironment, which blocked the activation of tumor-toxic T cells (14). For patients refused surgical interventions, PDT was an effective treatment approach without significant side effects (9). Both the direct cytotoxic and immunotherapy-synergetic effect of the PDT would contribute to its anti-tumor efficacy (15, 16). In this patient, the addition of PDT has synergized the efficacy of immunotherapy without bringing extra toxicities. The microscopic examinations of tumor biopsies pre-treatment and post-treatment have demonstrated the complete response. Comparing with the median 4.75 months from the treatment start to the clinical complete response in Asian MSI-H CRC patients receiving immunotherapy-based systemic therapy (5), the 42 days from the treatment start to the clinical complete response observed in this case was really impressive. The rapid as well as durable treatment response largely relied on the tumor-microenvironment-modulating effect of PDT, which remodeled the immunogenicity-scarce microenvironment to an immunotherapy-responsive microenvironment (15).

POLE mutation was seen in 9.8% of CRC patients ≤45 years compared to 1% in elder patients (17). POLE gene would translate to the largest subunit of DNA polymerase ε intracellularly, which was responsible for the proofreading fidelity of the polymerase in DNA replications (18). The mutation of POLE would cause the dysfunction of the polymerase and exonuclease domains of DNA Polymerase ε, which predisposed to various gastrointestinal malignancies (19). In the endometrial cancer and CRC, the pathogenic mutations of POLE were located at exon 9–14 while the mutation site in this patient was identified as A222C, at exon 7 (20, 21). Based on previous-published researches, the A222C mutations in this case has not been identified as pathogenic mutation, with unknown clinical relevance. CRC patients with POLE mutation were reported to have positive treatment responses with immune checkpoint inhibitors (22), partly because of the high CD4^+^ T cell infiltrations and increased expressions of pro-inflammatory cytokines in the tumor microenvironment (23). Moreover, the mutation of POLE would increase the tumor mutation burden by a high rate of base substitutions, which further enhanced the tumor immunogenicity (24). This case was diagnosed in his 40s, which was in accordance with the epidemic data in the previously-published study (23). Based on the abovementioned evidence, this patient was supposed to benefit from immunotherapy-based systemic therapies, while with the help of PDT locally, the anti-tumor effect was more durable compared to the clinical response reported in previous studies (22). The synergetic effect of PDT with immunotherapy was also due to the activations of the tumor immune microenvironment by PDT (25). The synergetic effect of both POLE-mutation-induced and PDT-induced tumor-antigen release would effectively activate the immune cells like CD4+ T cells, CD8+ T cells, and NK cells. The pre-activated immunologic effector cells were further potentiated by the use of PD-1 inhibitors, which translated to a reinforced anti-tumor effect and greater survival benefit (15, 25). Although the co-existence of POLE mutation has been frequently observed in CRC patients with MSI-H (26, 27), currently no studies have reported the efficacy of immunotherapy-based systemic therapy in CRC patients with both MSI-H and POLE mutations. Future results from ongoing clinical trials (NCT03150706, NCT03435107, NCT03810339, and NCT03827044) would validate the clinical benefit observed in this case.

Concerns about adverse events were critical factors in the decision-making process in the management of CRC, especially with combined therapeutic modalities (28). In this patient, the addition of PDT to the immunotherapy-based systemic therapy did not alter the safety profiles (**Figure 3**). Although with intensive anti-tumor treatments, the performance status of this patient has been satisfactory through these treatment cycles.

## Conclusion

In conclusion, we reported a young male synchronous CRC patient with MSI-H and POLE mutation had an accelerated and durable clinical response combining PDT with immunotherapy-based systemic therapies and has remained progression-free by far. This case highlighted the synergetic effect of PDT in immunotherapy-treated CRC patients with certain gene features such as MSI-H and POLE mutation. Future randomized clinical trials were warranted to clarify the role of PDT in CRC immunotherapy.

## Data availability statement

The raw data supporting the conclusions of this article will be made available by the authors, without undue reservation.

## Ethics statement

Written informed consent was obtained from the individual(s) for the publication of any potentially identifiable images or data included in this article.

## Author contributions

YW: Conceptualization, Data curation, Formal analysis, Funding acquisition, Investigation, Methodology, Project administration, Resources, Software, Supervision, Validation, Visualization, Writing – original draft, Writing – review & editing. LG: Data curation, Validation, Writing – original draft. BM: Data curation, Validation, Writing – review & editing. JS: Data curation, Visualization, Writing – review & editing. ZY: Data curation, Visualization, Writing – review & editing. WZ: Writing – review & editing. HC: Conceptualization, Funding acquisition, Project administration, Resources, Supervision, Writing – review & editing.
