# Supplementary material for: Accelerated clinical response achieved by combining short-term tumor-directed photodynamic therapy with immunotherapy-based systemic therapies in synchronous colorectal cancer with MSI-H and POLE mutation: a case report
Source: Front Immunol. 2024 Jun 28;15:1402334. doi: 10.3389/fimmu.2024.1402334 (PMC11239333; doi:10.3389/fimmu.2024.1402334)
Supplement: Supplementary file 1 [file Table_1.docx]

Supplementary Material

Accelerated clinical remission achieved by combining tumor-directed photodynamic therapy with immunotherapy-based systemic therapies in synchronous colorectal cancer with MSI-H and POLE mutation: a case report.

**Yuhan Wang^1^, Lei Gao^1^, Bin Ma^1^, Jianming Shi^1^, Zhenyu Yin^1^, Weidong Zhu^2^, Hao Chen^3^**

*** Correspondence:** Weidong Zhu. [35868971@qq.com](mailto:35868971@qq.com). Hao Chen. [ery_chenh@lzu.edu.cn](mailto:ery_chenh@lzu.edu.cn).

Keywords: Colorectal Neoplasms; Immunotherapy; Photodynamic Therapy; Microsatellite Instability; POLE protein; human.

**Table 1. Next generation sequencing results of tumor biopsy.**

| Treatment-related gene mutations | Considerations for clinical management |
| --- | --- |
| Microsatellite-instability status: High. | Immunotherapy  (e.g. PD-1 inhibitor) |
| Tumor mutational burden level: High  (72.7 mutations/Mb). | Immunotherapy  (e.g. PD-1 inhibitor) |
| POLE : E7 mutation detected (p.Arg222Cys). | Immunotherapy  (e.g. PD-1 inhibitor) |
| POLD1 : E2 and E11 mutations detected (p.Pro15fs, p.Ser429Ala). | Immunotherapy  (e.g. PD-1 inhibitor) |
| PD-L1 : negative. |  |
| KRAS E2 mutation detected (p.Gly12Val). | NA |
| PIK3CA E10 mutation detected (p.Glu542Lys). | NA |
| APC: E8, E15, E16, E18 mutations detected (p.Ala272Thr, p.Ser587fs, p.Gln1090*, p.Thr1556fs, p.Glu1080*, p.Gly2362*, p.Ile679fs). | NA |
| ARID1A : E18 mutation detected (p.Gln1519fs). | NA |
| BRAF : no mutation detected. |  |
| HER2 : no mutation detected. |  |
| **Germline and somatic mutations indicating Lynch syndrome or hereditary colorectal cancer** (genes: EPCAM、MLH1、MSH2、MSH6、PMS2): no mutations detected. |  |

NA: not applicable.

**2. Expressions of PD-L1 in the tumor sample of this patient**


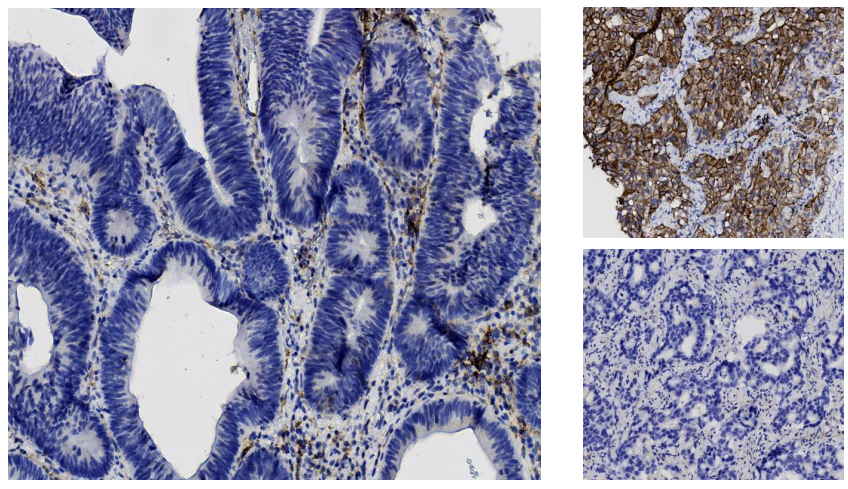


The left picture was the expressions of PD-L1 in the tumor sample of this patient using immunohistochemistry technique. The right pictures were the expressions of PD-L1 of the positive and negative control samples using immunohistochemistry technique.

**2. Details of the PDT process**

The PDT was conducted as follow: after tested negative for the photosensitizer allergy, the patient was infused with hematoporphyrin (3mg/kg, Milelonge Biopharmaceutical Co., LTD, China) before PDT treatment. The patient was then protected from the sunlight to avoid skin reactions. Endoscopic irradiation by the 5cm-laser-device (Xingda Photoelectric Medical Instrument Co., LTD, China) was initiated 36 hours post-infusion. The irradiation parameters were as follows: wavelength was set to be 630 nm, power was set to be 800 mW, and energy density was set to be 200 J/cm^2^. Each photodynamic irradiation would last for 5-10 minutes. Intensive light protection was adopted for the patient post-irradiation.
